# Large expansion of CTG•CAG repeats is exacerbated by MutSβ in human cells

**DOI:** 10.1038/srep11020

**Published:** 2015-06-05

**Authors:** Rie Nakatani, Masayuki Nakamori, Harutoshi Fujimura, Hideki Mochizuki, Masanori P. Takahashi

**Affiliations:** 1 Department of Neurology, Osaka University Graduate School of Medicine; 2 Department of Neurology, Toneyama Hospital, National Hospital Organization.

## Abstract

Trinucleotide repeat expansion disorders (TRED) are caused by genomic expansions of trinucleotide repeats, such as CTG and CAG. These expanded repeats are unstable in germline and somatic cells, with potential consequences for disease severity. Previous studies have demonstrated the involvement of DNA repair proteins in repeat instability, although the key factors affecting large repeat expansion and contraction are unclear. Here we investigated these factors in a human cell model harboring 800 CTG•CAG repeats by individually knocking down various DNA repair proteins using short interfering RNA. Knockdown of MSH2 and MSH3, which form the MutSβ heterodimer and function in mismatch repair, suppressed large repeat expansions, whereas knockdown of MSH6, which forms the MutSα heterodimer with MSH2, promoted large expansions exceeding 200 repeats by compensatory increases in MSH3 and the MutSβ complex. Knockdown of topoisomerase 1 (TOP1) and TDP1, which are involved in single-strand break repair, enhanced large repeat contractions. Furthermore, knockdown of senataxin, an RNA/DNA helicase which affects DNA:RNA hybrid formation and transcription-coupled nucleotide excision repair, exacerbated repeat instability in both directions. These results indicate that DNA repair factors, such as MutSβ play important roles in large repeat expansion and contraction, and can be an excellent therapeutic target for TRED.

More than 20 human neurodegenerative diseases are caused by trinucleotide repeat expansions in genomic DNA, including Huntington disease (HD) and several forms of spinocerebellar ataxia (SCA; CAG expansions), as well as myotonic dystrophy type 1 (DM1; CTG expansions) [reviewed by^1^]. In these trinucleotide repeat expansion disorders (TRED), the mutations are unstable, and exhibit an exceptional degree of genetic instability in germinal cells. Because the repeat lengths correlate with the age of onset and disease severity, the tendency of unstable repeats to expand in the germline can lead to marked phenotypic anticipation within families. Instability of expanded repeats also occurs in somatic cells throughout life, and this may affect the age of symptom onset or the rate of disease progression. Considerable evidence has suggested that expanded trinucleotide repeat instability is associated with DNA metabolizing processes such as replication, repair, or transcription^1^. Previous studies on bacteria and yeast have shown that many trans-factors involved in these processes affect repeat instability. Studies on animal models have greatly improved our understanding of the roles of trans-factors in repeat instability *in vivo*. In particular, the importance of mismatch repair (MMR) proteins was demonstrated when DM1 or HD mouse models were crossed with key DNA repair enzyme-knockout mouse models^1^. Mammalian MMR proteins include MSH2 (MutS homolog 2), MSH3 (MutS homolog 3), and MSH6 (MutS homolog 6), which form the heterodimers MutSα (MSH2/MSH6) and MutSβ (MSH2/MSH3)^2^. However, the detailed roles of the individual factors remain obscure or even controversial. For example, ablation of MSH2 or MSH6 reportedly suppresses CTG repeat instability in some models^3^,^4^,^5^ and enhances repeat instability in other models^6^,^7^. This discrepancy may be due to the complexity of the MMR system *in vivo* or the involvement of other factors such as chromatin modification and transcriptional activities. To dissect the role of each factor with regard to repeat instability, cell models can be a suitable system, with the advantages of having less complexity, a shorter duration to observe repeat size changes, and easy modulation of trans-factors. Lin *et al*.^8^ recently established an excellent cell model harboring 95 CAG repeats to study the role of trans-factors in repeat instability. This model could selectively detect the frequency of repeat contraction events through transcription of the repeat. By incorporating short interfering RNA (siRNA), they revealed the involvement of various DNA repair factors in transcription-coupled repeat contraction^8^,^9^,^10^,^11^. In addition, similar knockdown by siRNA in other cell models showed the implication of MMR in small incremental expansions of CTG•CAG repeats^12^,^13^. However, the factor that regulates the large repeat expansion that contributes to genetic anticipation and disease progression in TRED remains unknown. We previously established a human cell model, HT1080-800R, which reproduced both the expansion and contraction modes of CTG•CAG repeat instability^14^. With this model, we were able to monitor the instability of large CTG•CAG repeats, involved in DNA replication, repair, and transcription for a period of 1 month. To elucidate the individual role of each trans-factor, we induced sustained knockdown using siRNA and revealed the essential factors affecting large repeat expansion or contraction and possible important therapeutic targets for preventing disease progression in TRED.

## Materials and methods

### Cell culture

The construction of the HT1080-800R cell model has been described previously^14^. In brief, HT1080 human fibrosarcoma cells were cotransfected with a plasmid (LC15-F) containing 800 CTG•CAG repeats^15^ and a plasmid encoding PhiC31 integrase. Transfection was performed with a Nucleofector (Lonza, Basel, Switzerland), and stably transfected clones were selected with puromycin. For siRNA treatment, the HT1080-800R cells were plated in a 96-well plate, and 1-μM (manufacturer’s recommended concentration for effective target knockdown and less off-target effects) Accell SMARTpool siRNA (GE Healthcare, Pittsburgh, PA) was added 6 h later. Cells were incubated at 37 °C with 5% CO_2_ in Accell siRNA Delivery Media (GE Healthcare) with 2% fetal bovine serum. Cells were passaged twice weekly with continuous exposure to 1-μM siRNA. An Accell Non-targeting Control Pool (GE Healthcare) was used as a control in all siRNA transfection experiments.

### Human brain samples

Human brain samples (temporal cortex and cerebellum) were obtained via autopsy from three DM1 patients following informed consent from patients’ family. All experimental protocols were approved by the Institutional Review Board at Osaka University, and carried out in accordance with the approved guidelines. The repeat sizes in those samples have been described elsewhere^16^. All temporal cortex samples had >1900 CTG repeats with a high somatic heterogeneity, whereas cerebellar samples had <300 repeats.

### Repeat length analysis

Genomic DNA was extracted from HT1080-800R clones using the Gentra Puregene Cell Kit (Qiagen, Valencia, CA). The expanded CTG repeats were sized by small-pool PCR followed by Southern blot as described previously^15^. At least 50 alleles were analyzed for each group^14^.

### mRNA quantification

RNA was harvested from the HT1080-800R cells at 72 h after adding siRNA using the RNeasy Micro Kit (Qiagen). RNA was prepared from human brain samples as described previously^17^. Total RNA was primed with random hexamers and reverse transcribed with Superscript III (Life Technologies, Carlsbad, CA), followed by treatment with RNase H. Quantitative reverse transcription (RT)–PCR was performed using TaqMan Gene Expression assays on an ABI PRISM 7900HT Sequence Detection System (Life Technologies). Relative expression was calculated using delta-delta Ct method.

### Protein analysis

HT1080-800R cells were washed in phosphate-buffered saline (PBS) and lysed in M-PER Mammalian Protein Extraction Reagent (Thermo Scientific) supplemented with Protease Inhibitor Cocktail (Sigma–Aldrich, St. Louis, MO) at 72 h after siRNA addition to extract proteins. Proteins were extracted from human brain samples by mechanical homogenization in lysis buffer [0.125-M, Tris-HCl (pH 6.8), 4% sodium dodecyl sulfate (SDS), 10% glycerol] containing Protease Inhibitor Cocktail (Sigma–Aldrich). SDS polyacrylamide gel electrophoresis (SDS-PAGE) was performed as described previously^17^. Blots were blocked with 5% (weight/volume) nonfat milk and then incubated with antibodies at the following dilutions: anti-MSH2 (FE11; Life Technologies), 1:500; anti-MSH3 (611390; BD Biosciences, San Jose, CA), 1:200; anti-MSH6 (610919; BD Biosciences), 1:1000; and anti-beta-actin (WAKO, Saitama, Japan), 1:1000. After repeated washings, the membranes were incubated with horseradish peroxidase-conjugated goat anti-mouse IgG (Life Technologies). The ECL Plus western blotting detection system (GE Healthcare) and a luminescent image analyzer (ImageQuant LAS-4000, GE Healthcare) were used to detect the proteins.

For the immunoprecipitation (IP) analysis, whole-cell lysates were centrifuged for 10 min at 15,000 × *g*. The supernatants were then incubated with anti-MSH2, followed by overnight incubation with Dynabeads Protein G (Life Technologies). The beads were then washed, and bound proteins were separated via SDS-PAGE and analyzed by immunoblotting with anti-MSH3.

### Chromatin immunoprecipitation

Chromatin immunoprecipitation (ChIP) assays were performed using an EZ-ChIP kit (Merck Millipore) according to the manufacturer’s instructions. Briefly, at 72 h after the addition of siRNA, the cells were first cross-linked with 1.5-mM dithiobis-succinimidyl propionate, followed by 1% formaldehyde. Cell lysates were sonicated for 180 s (Branson sonifier 250, setting 1) to yield chromatin fragments of approximately 1000 bp. IP reactions were set up with 5 μg each of MSH2 (ab16833, Abcam) or MSH3 antibody (ab74607, Abcam); normal mouse IgG was used as a negative control to assess levels of background. The quantitative PCR-based analysis with primers specific for the region downstream of the CTG repeats was performed as described previously^14^.

### Statistics

For repeat length analysis, χ^2^-tests were performed to compare the frequencies of expanded, unchanged, and contracted alleles in each set of experiments as reported previously^14^. Paired t-tests were performed for expression analysis of mRNA and protein.

## Result

### Continuous siRNA knockdown of trans-factors in the HT1080-800R model

**Figure 1:**
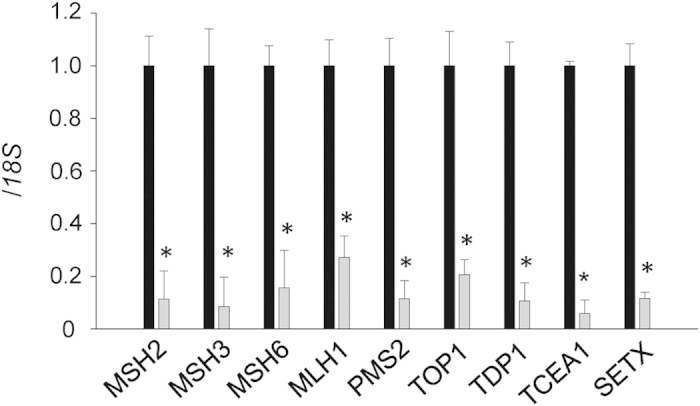
Efficiency of the siRNA knockdown targeting each trans-factor. RNA levels in siRNA-treated HT1080-800R cells were determined via quantitative reverse transcriptase PCR and normalized to *18S* rRNA. The expression of each target was reduced by sustained specific siRNA knockdown (gray bars) when compared with the expression in cells treated with the non-targeting control siRNA (black bars). Data are presented as means ± standard deviations (SD) of quadruplicate experiments. *P < 0.001.

Our cell model, HT1080-800R, contains 800 CTG•CAG repeats with transcription in the CUG-repeat direction^14^. Previously, we demonstrated progressive repeat instability in this model during a 1-month culture period based on DNA replication, repair, and transcription. Herein, we used this model to investigate the individual trans-factors implicated in repeat instability via continuous siRNA knockdown for a period of 1 month. We knocked down nine trans-factors involved in MMR, transcription-coupled nucleotide excision repair (TC-NER), or single-strand break repair (SSBR). Each siRNA treatment significantly reduced the target expression to <27% (Fig. 1). However, siRNA treatments that affect cell proliferation may also affect the DNA replication rate and thereby influence repeat instability. To ensure that sustained siRNA treatment did not affect proliferation, we calculated the growth rates of the HT1080-800R cells in each siRNA treatment and found no effects from any of the siRNA treatments (Supplemental Figure S1).

### DNA repair factors involved in MMR, SSBR, and TC-NER regulate large repeat expansion and contraction

**Figure 2:**
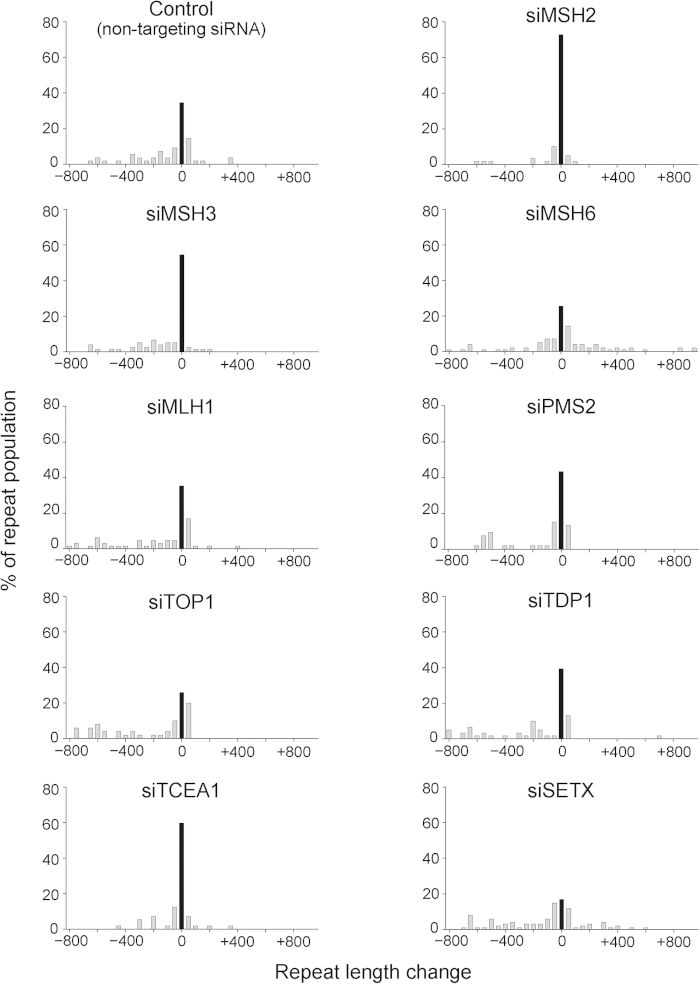
Effects of sustained trans-factor knockdown on CTG•CAG repeat instability in HT1080-800R cells. Repeat instability was analyzed by small-pool PCR followed by Southern blotting. Histograms show the repeat-length distributions in the HT1080-800R cells treated with each siRNA. The frequency distribution of unstable alleles is indicated as gray bars. The frequency of stable alleles is indicated as black bars. Allele lengths are grouped in bins spanning 50 repeats. More than 50 alleles were sized per group.

MMR proteins have been extensively studied to elucidate the mechanism of repeat instability. MSH2 was especially suggested as a potential promoter of repeat instability both *in vitro* and *in vivo*^1^. In our study, sustained MSH2 knockdown significantly reduced both the expansion and contraction modes of repeat instability and eliminated “big jumps” involving expansions of several hundred repeats (Fig. 2 and Supplemental Figure S2). The cumulative frequency of unstable alleles in siMSH2-treated cells was 27.1% (6.8% expansions and 20.3% contractions), whereas that in control-treated cells was 65.4% (21.8% expansions and 43.6% contractions; Table 1). Similarly, knockdown of MSH3, which forms MutSβ with MSH2, also reduced repeat instability, especially expansions (6.5% expansion versus 54.5% unchanged versus 39.0% contraction for all alleles; Fig. 2 and Table 1). Previous cell model-based studies reported that knockdown of MSH6, which forms MutSα with MSH2, did not affect repeat instability^8^,^12^,^18^. However, in our study, MSH6 knockdown strongly enhanced repeat instability with a bias toward expansion (41.8% expansion versus 25.5% unchanged versus 32.7% contraction for all alleles; Fig. 2 and Table 1). Many alleles exhibited changes of >200 repeats and some alleles gained >1000-CTG repeat expansions (average change in the repeat size: +21 CTG repeats versus −90 in the control). In contrast, knockdown of MLH1 or PMS2, which form the MutLα heterodimer and act downstream of MutS homologue mismatch recognition, did not affect repeat instability.

We also tested two components, topoisomerase 1 (TOP1) and tyrosyl-DNA phosphodiesterase 1 (TDP1), which have been implicated in the SSBR pathway^19^,^20^. A previous study showed that inhibition of the TOP1-TDP1-SSBR pathway led to an increased frequency of repeat contractions^11^. In our study, although neither TOP1 nor TDP1 knockdown significantly affected repeat instability (Fig. 2), the average change in the repeat size was biased toward contraction (−204.7 and −172.7 CTG repeats, respectively, versus −89.7 in the control; Table 1). Then, we further studied the cumulative effects by simultaneous knockdown of TOP1 and TDP1. The double knockdown, which resulted in reductions in target transcript expression (Supplemental Figure S3A), significantly promoted large repeat contraction, compared to single knockdowns of each factor (8.4% expansion versus 19.3% unchanged versus 72.3% contraction for all alleles; Supplemental Figure S3B and Table 1).

Recent evidence has suggested that somatic instability in expanded repeats is associated with TC-NER^9^. In the TC-NER pathway, transcription elongation factors reportedly play a crucial role by modulating trafficking of RNA polymerase II^21^. In addition, DNA:RNA hybrids called R-loops, suggested to be processed in the TC-NER pathway, block transcription elongation and induce transcription-coupled repeat instability^22^,^23^. Indeed, inhibition of R-loop formation was reported to reduce repeat instability *in vitro* and *in vivo*^14^. In this study, we knocked down *TCEA1* which encodes the transcription elongation factors IIS, and senataxin (SETX), an RNA:DNA helicase that resolves R-loops formation^24^. The knockdown of *TCEA1* reduced both the expansion and contraction modes of repeat instability (12.3% expansion versus 59.6% unchanged versus 28.1% contraction for all alleles; Fig. 2 and Table 1). In contrast, knockdown of SETX significantly enhanced repeat instability in both directions (26.7% expansion versus 16.8% unchanged versus 56.4% contraction for all alleles; Fig. 2 and Table 1). These results indicate the implication of transcription-coupled repair factors in large repeat size changes.

### MutSβ enhances repeat expansion

**Figure 3:**
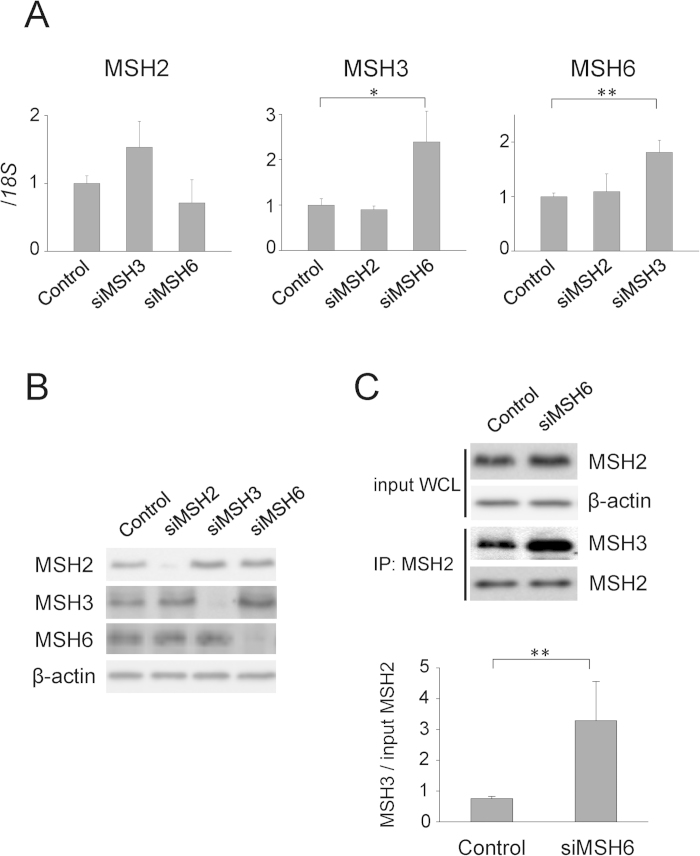
(**A**) Expression levels of MutS homologues genes (*MSH2*, *MSH3*, and *MSH6*) following each siRNA treatment as determined by *18S* rRNA-normalized quantitative reverse transcription PCR. Data are presented as means ± standard deviations (SD) of quadruplicate experiments. *P < 0.01, **P < 0.05. (**B**) Representative immunoblots of MSH2, MSH3, and MSH6 protein expressions in HT1080-800R cells following the siRNA treatment. Beta-actin was used as a loading control. The gels were run under the same experimental conditions. (**C**) *Top*: MSH3 immunoprecipitation (IP) with an anti-MSH2 antibody and MSH2 and β-actin immunoblot of whole-cell lysates (WCL) used in IP following the siRNA treatment. *Bottom*: Relative amounts of MSH3 immunoprecipitated with an anti-MSH2 antibody. Data are presented as means ± SD of triplicate experiments. *P < 0.05.

In previous reports, MSH6 knockdown did not affect the frequency of repeat contraction in human cell models^8^,^18^. Similarly in our model, MSH6 knockdown did not increase repeat contraction but did significantly exaggerate repeat expansion. As MSH2 knockdown did not promote repeat instability, it is unlikely that a decrease in the MutSα complex induced this repeat expansion. Previous studies indicated that MSH6 knockdown induces compensatory MSH3 overexpression and vice versa, because MSH6 and MSH3 compete for MSH2 binding^25^,^26^. To study the compensatory upregulation of these factors, we performed quantitative RT-PCR after each siRNA treatment, and this resulted in increased *MSH3* and *MSH6* expressions (Fig. 3A). Immunoblotting revealed that MutS homologue knockdown led to significant reductions in target protein expression (MSH2, 7.5 ± 5.6%; MSH3, 9.6 ± 1.1%; MSH6, 12.1 ± 11.4%; Fig. 3B and Supplemental Figure S4). Similar to the mRNA findings, MSH6 knockdown induced a compensatory increase in MSH3 expression (2.4-fold), although MSH3 knockdown did not increase MSH6 protein expression (Supplemental Figure S4). To determine whether the MutSβ complex formation increased after the siMSH6 treatment, we performed immunoprecipitation assays with anti-MSH2 antibody. The amount of MSH3 pulled down by the anti-MSH2 antibody was significantly higher in the siMSH6-treated cells compared with that in the control-treated cells, indicating a compensatory increase in the MutSβ complex formation following MSH6 knockdown (Fig. 3C).

**Figure 4:**
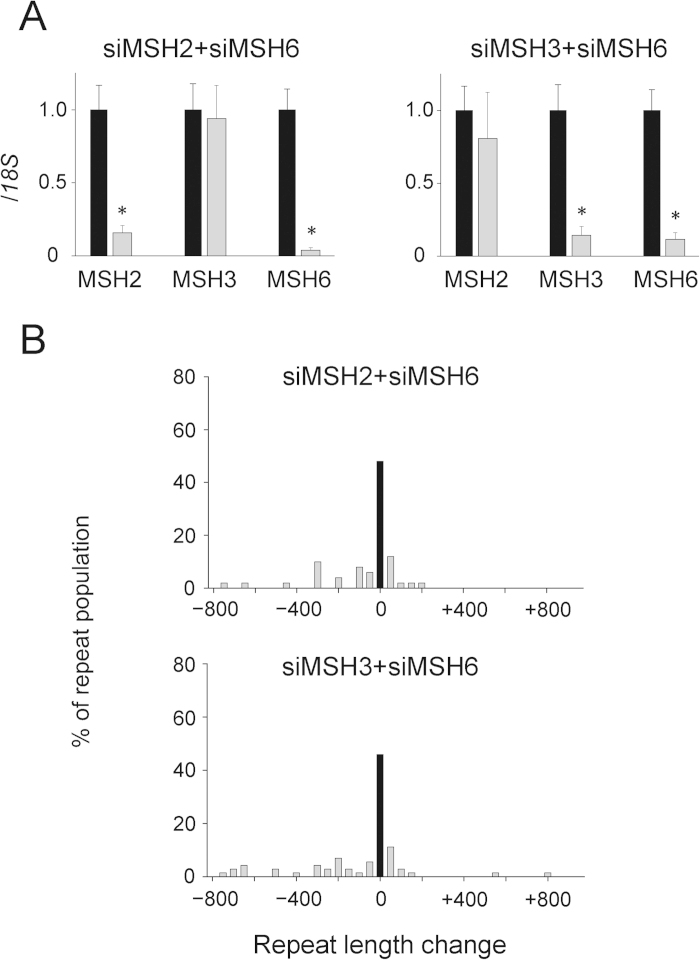
(**A**) Expression levels of MutS homologue genes (*MSH2*, *MSH3*, and *MSH6*) following double siRNA knockdown as determined by *18S* rRNA-normalized quantitative reverse transcription PCR. The expression of each target was reduced by sustained specific siRNA knockdown (gray bars) when compared with the expression in cells treated with the non-targeting control siRNA (black bars). Data are presented as means ± standard deviations (SD) of triplicate experiments. *P < 0.001. (**B**) Effects of double MSH2 and MSH6 (MSH2&MSH6) or MSH3 and MSH6 (MSH3&MSH6) knockdown on CTG•CAG repeat instability in HT1080-800R cells. Histograms show the repeat-length distributions in HT1080-800R cells. The frequency distribution of unstable alleles is indicated as gray bars. The frequency of stable alleles is indicated as black bars. Allele lengths are grouped in bins spanning 50 repeats. More than 50 alleles were sized per group.

Next, to investigate whether the compensatory increase in MutSβ resulting from MSH6 knockdown would affect repeat instability, we evaluated the effect of a simultaneous MSH6 and MSH2 or MSH3 knockdown. Double MSH2 and MSH6 or MSH3 and MSH6 knockdown resulted in significant reductions in target transcript expression but did not affect the proliferation rates (Fig. 4A and Supplemental Figure S1). Repeat instability was analyzed after a 1-month sustained double knockdown treatment, and the results were compared with those of the single MSH6 knockdown. Simultaneous MSH6 and MSH2 or MSH3 knockdown, which offset the compensatory increase in MutSβ, significantly reduced repeat instability when compared with the single MSH6 knockdown (Fig. 4B versus siMSH6 in Fig. 2; Table 1), indicating a pivotal role of MutSβ in repeat expansion. Furthermore, to determine whether the increased MutSβ following the siMSH6 treatment was recruited to the expanded CTG repeats, we performed ChIP assays with anti-MSH2 or anti-MSH3 antibody. The results indicated that both MSH2 and MSH3 localizations were significantly enriched just downstream of the CTG repeats in siMSH6-treated HT1080-800R cells compared with those treated with the non-targeting control siRNA (Supplemental Figure S5).

### MMR proteins in the DM1 brain

**Figure 5:**
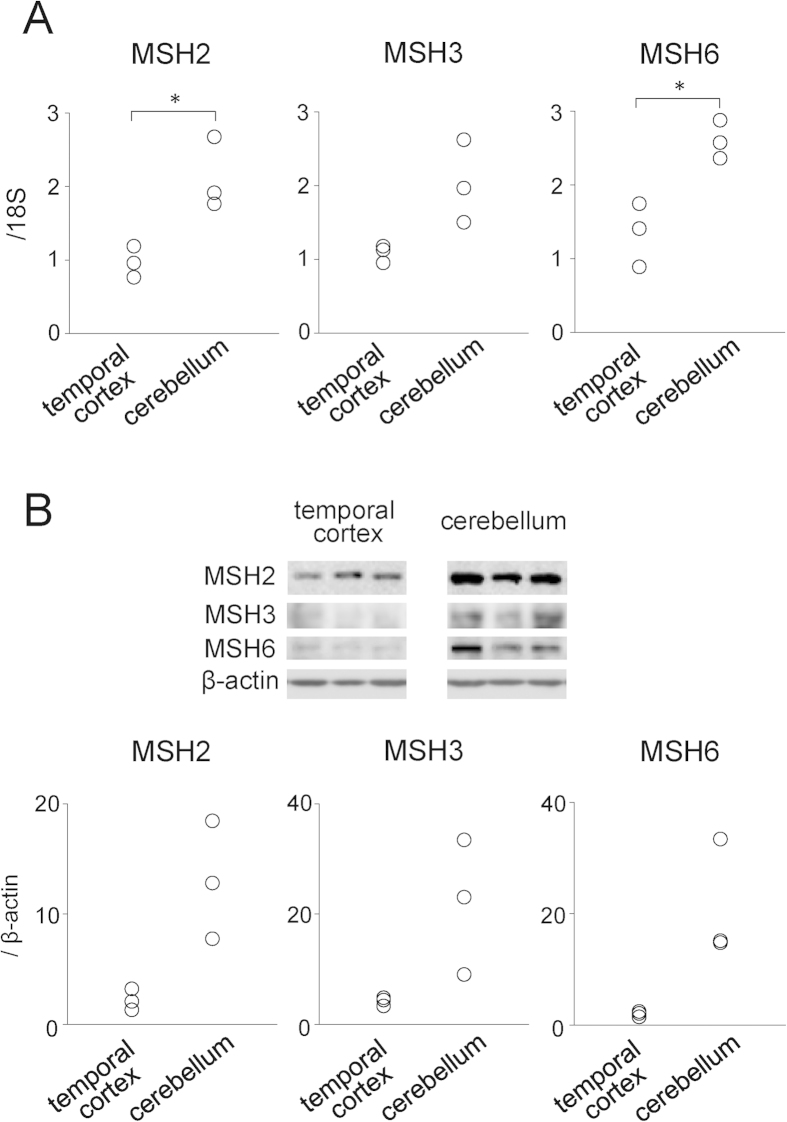
(**A**) Expression levels of MutS homologue genes (*MSH2*, *MSH3*, and *MSH6*) in DM1 brain tissues as determined by *18S* rRNA-normalized quantitative reverse transcription PCR. *P < 0.05. (**B**) *Top*: Representative immunoblots of MSH2, MSH3, and MSH6 protein expressions in the DM1 brain. β-actin was used as a loading control. The immunoblot signals of temporal cortex and cerebellum were assessed on the same immunoblots and under the same exposure conditions. *Bottom*: Scatter plot of MMR protein expression in brain tissues of three DM1 patients.

A characteristic feature of TRED, and particularly DM1, is somatic instability of the expanded repeat^27^. This marked instability leads to 10-fold variations in the expansion lengths among different tissues in a DM1 individual^28^,^29^,^30^,^31^. Typical DM1 patients harbor several thousand repeats in the skeletal muscle, heart, and cerebral cortex but several hundred repeats in the leukocytes and cerebellum^31^. Because the results from the HT1080-800R cell model indicated that MutSβ strongly promoted repeat instability, one can imagine that MutSβ is abundantly expressed in tissues with higher levels of somatic instability. However, previous studies showed a negative correlation between MMR protein expression and levels of repeat instability in the striatum and cerebellum in HD patients and various HD model mice^32^,^33^,^34^. To investigate a possible relationship between the somatic instability in DM1 and the expression levels of MutS homologue proteins, we examined the mRNA and protein expression levels of MSH2, MSH3, and MSH6 in the DM1 brain. We compared the expression in the cerebral (temporal) cortex and cerebellum in three DM1 patients. However, as is the case with the negative correlation in HD, the mRNA levels of *MSH2, MSH3*, and *MSH6* were lower in the temporal cortex than those in the cerebellum from DM1 patients (Fig. 5A). In addition, immunoblotting revealed lower protein levels in the temporal cortex (Fig. 5B). We also performed immunohistochemistry to study cellular distribution of MSH proteins in human brain (temporal cortex). MSH2, MSH3, and MSH6 proteins were detected in the neuronal cells, but not in the majority of non-neuronal cells (Supplemental Figure S6), similar to a previous study reporting cellular distribution of MSH3 in human striatum^35^. Although the MutSα and MutSβ ratios remained uncertain because of the difficulties in the immunoprecipitation analysis of brain tissues, the results implicate other factors in the somatic instability within the DM1 brain, such as cell metabolism, as suggested previously^32^.

## Discussion

In this study, we demonstrated the importance of MutSβ with respect to large repeat expansion using a simple knockdown method in our human cell model. The knockdown of MSH2 and MSH3, the components of MutSβ, both stabilized the highly expanded (CTG•CAG)_800_ repeats and reduced large expansion gains. In previous studies of other cell models, knockdown of either the MutSβ protein led to the stabilization of (CAG)_95_, (CTG)_22_, and (GAA)_176_ repeats^8^,^12^,^18^. These results indicate that MutSβ enhances the repeat instability of both short and long expansion as well as different repeat motifs. We also found that MSH6 knockdown promoted repeat instability, especially large expansions involving gains of >1000 CTG•CAG repeats. The average change in the repeat size following MSH6 knockdown was >100 CTG repeats greater than those in the control. Furthermore, MSH6 knockdown increased the number of MSH3 and MutSβ complexes and MutSβ enrichment around the expanded CTG tract. The exacerbated repeat instability following MSH6 knockdown was presumably due to MutSβ upregulation rather than MutSα downregulation because (1) MSH2 knockdown did not promote repeat expansion, (2) double MSH3 and MSH6 knockdown reduced repeat instability, and (3) functional redundancies in the MMR system can compensate for MSH6 depletion by shifting MSH2 dimerization to MSH3 to form MutSβ. In theory, MutSα recognizes base–base mismatches and insertion/deletion loops of 1–3 nucleotides, whereas MutSβ binds insertion/deletion loops of 1–12 nucleotides^36^. These different DNA mismatch-recognition specificities may explain the reason for CTG•CAG repeat instability being oppositely affected by MSH3 and MSH6 downregulation in our model. In previous studies of other cell models, MSH6 knockdown did not affect repeat instability^8^,^12^,^18^. The reason for the discrepancy with our study is unclear, but it may be related to different motifs, repeat sizes, or cell types. Nonetheless, our study supports a previous observation that MSH6-deficient mice, in which only MutSβ complexes are formed, exhibited increased somatic instability in expanded CTG repeats^7^.

We also demonstrated the effect of other trans-factors on repeat instability. Double knockdown of TOP1 and TDP1, which are involved in SSBR, promoted large repeat contraction. Hubert *et al*. previously reported that siRNA-mediated knockdowns of each factor increased frequency of repeat contractions, and proposed the TOP1-TDP1-SSBR pathway for repeat instability^11^. The enhanced repeat contraction by pairwise knockdown of TOP1 and TDP1 in our study indicates a possibility that both factors independently regulate repeat instability. In a previous study, knockdown of TCEA1, a transcription elongation factor, reduced the frequency of contraction events of (CAG)_95_ repeats^9^. Our study also showed reductions in both the expansion and contraction of (CTG)_800_ repeats following TCEA1 knockdown. In addition, knockdown of SETX, which suppresses R-loops formation and regulates transcription, enhanced repeat instability in both directions. These results reinforce the hypothesis that transcriptional elongation plays a pivotal role in repeat instability^37^. In our study, the knockdown of neither PMS1 nor MLH1 affected repeat instability, whereas a previous report showed increases of contraction frequency by siRNA-mediated depletion of PMS1 or MLH1^10^. To observe significant effects, it may be necessary to achieve a severe reduction or complete depletion of these factors. Therefore, we cannot exclude the possibility that these factors are involved in repeat instability.

Although our study strengthens the importance of MMR in repeat instability regulation, MMR protein expression was not directly related to somatic instability in the DM1 brain. In DM1 and other TRED, the cerebellum consistently displays the shortest expansion size and most limited length heterogeneity^31^. However, the MSH2, MSH3, and MSH6 protein expression levels were higher in the DM1 cerebellum than in the temporal cortex, which harbors 10-fold larger repeats. Similar results have been observed in the striatum and cerebellum in both HD patients and model mice^32^,^33^,^34^. One possible explanation is that factors other than the MMR protein level, such as transcription activity or epigenetic changes, contribute to repeat instability. Alternatively, because increased MutSβ levels resulted in “big jumps” of expanded repeats, the MMR proteins affect intergenerational instability that leads to large repeat expansions in the offspring, resulting in an earlier age of onset, rather than somatic instability. The distribution of expanded alleles followed by MSH6 knockdown implies salutatory changes seen in intergenerational anticipation, rather than gradual increases due to the accumulation of many small changes, or rapid cell growth with larger repeats (i.e., not biphasic) (Fig. 2).

Because ongoing repeat expansion is considered to contribute to disease progression in TRED, stabilization of the repeat could postpone the onset or slow progression^14^. Our study has also provided a possible therapeutic target for stabilizing expanded repeats. Because we demonstrated that MutSβ exacerbates repeat expansions, MutSβ disruption is a reasonable therapeutic approach. Since MSH3 deficiencies, unlike MSH2 deficiencies, do not increase genome-wide instability or cancer risks, MSH3 downregulation is a good target to stabilize the repeat, as proposed in previous reports^1^,^18^,^38^. In addition, because knockdown of TCEA1 or SETX also affected repeat instability, modulation of transcriptional elongation can be another therapeutic approach. Thus, our cell model is an appropriate tool for identifying potential therapeutic targets by testing the effects of individual knockdowns of trans-factors involved in transcription-induced repeat instability.

## Additional Information

**How to cite this article**: Nakatani, R. *et al*. Large expansion of CTG·CAG repeats is exacerbated by MutSβ in human cells. *Sci. Rep*. **5**, 11020; doi: 10.1038/srep11020 (2015).

## Supplementary Material

Supplementary Information

## Figures and Tables

**Table 1:** Effects of trans-factor knockdown on repeat instability in HT1080-800R cells.

Target	% expansion^a^	% unchanged	% contraction^a^	P value^b^	Avg change of repeat size^c^
Control (non-targeting siRNA)	21.8	34.5	43.6		−89.7
siMSH2	6.8	72.9	20.3	1.87E-04	−35.4
siMSH3	6.5	54.5	39.0	1.23E-02	−95.6
siMSH6	41.8	25.5	32.7	4.42E-02	21.2
siMLH1	21.5	35.4	43.1	1.00	−140.1
siPMS2	13.2	43.4	43.4	0.43	−125.3
siTOP1	20.0	26.0	54.0	0.54	−204.7
siTDP1	14.8	39.3	45.9	0.60	−172.7
siTCEA1	12.3	59.6	28.1	2.83E-02	−30.4
siSETX	26.7	16.8	56.4	0.04	−117.8
	% expansion^a^	% unchanged	% contraction^a^	P value^d^	Avg change of repeat size^c^
siMSH2 & siMSH6	18.0	48.0	34.0	4.88E-03	−70.7
siMSH3 & siMSH6	18.1	45.8	36.1	1.87E-03	−91.6
	% expansion^a^	% unchanged	% contraction^a^	P value^e^	Avg change of repeat size^c^
siTOP1 & siTDP1	8.4	19.3	72.3	< 0.05	−254.7

^a^A cut-off point of ±25 repeats was used to determine expansion and contraction.

^b^P-values were calculated using the χ^2^ test to compare the proportions of expanded, unchanged, and contracted alleles within the populations of target siRNA-treated versus control-treated cells.

^c^For all alleles (expanded + unchanged + contracted), the average change in the repeat size is expressed as the number of repeats. Note that the average change in the repeat size was biased toward contraction because of the preferential amplification of shorter alleles by small pool PCR.

^d^P-values were calculated using the χ^2^ test to compare the proportions of expanded, unchanged, and contracted alleles in the populations of double siRNA-treated versus siMSH6-treated cells.

^e^P-values were calculated using the χ^2^ test with Holm’s correction to compare the proportions of expanded, unchanged, and contracted alleles in the populations of double siRNA-treated versus siTOP1- or siTDP1-treated cells.
