# Supplementary material for: Large expansion of CTG•CAG repeats is exacerbated by MutSβ in human cells
Source: Sci Rep. 2015 Jun 5;5:11020. doi: 10.1038/srep11020 (PMC4457148; doi:10.1038/srep11020)
Supplement: Supplementary Information [file srep11020-s1.pdf]

# Supplementary Information

## Title: Large expansion of CTG•CAG repeats is exacerbated by MutSβ in human cells

Author: Rie Nakatani, Masayuki Nakamori, Harutoshi Fujimura, Hideki Mochizuki, and Masanori P. Takahashi

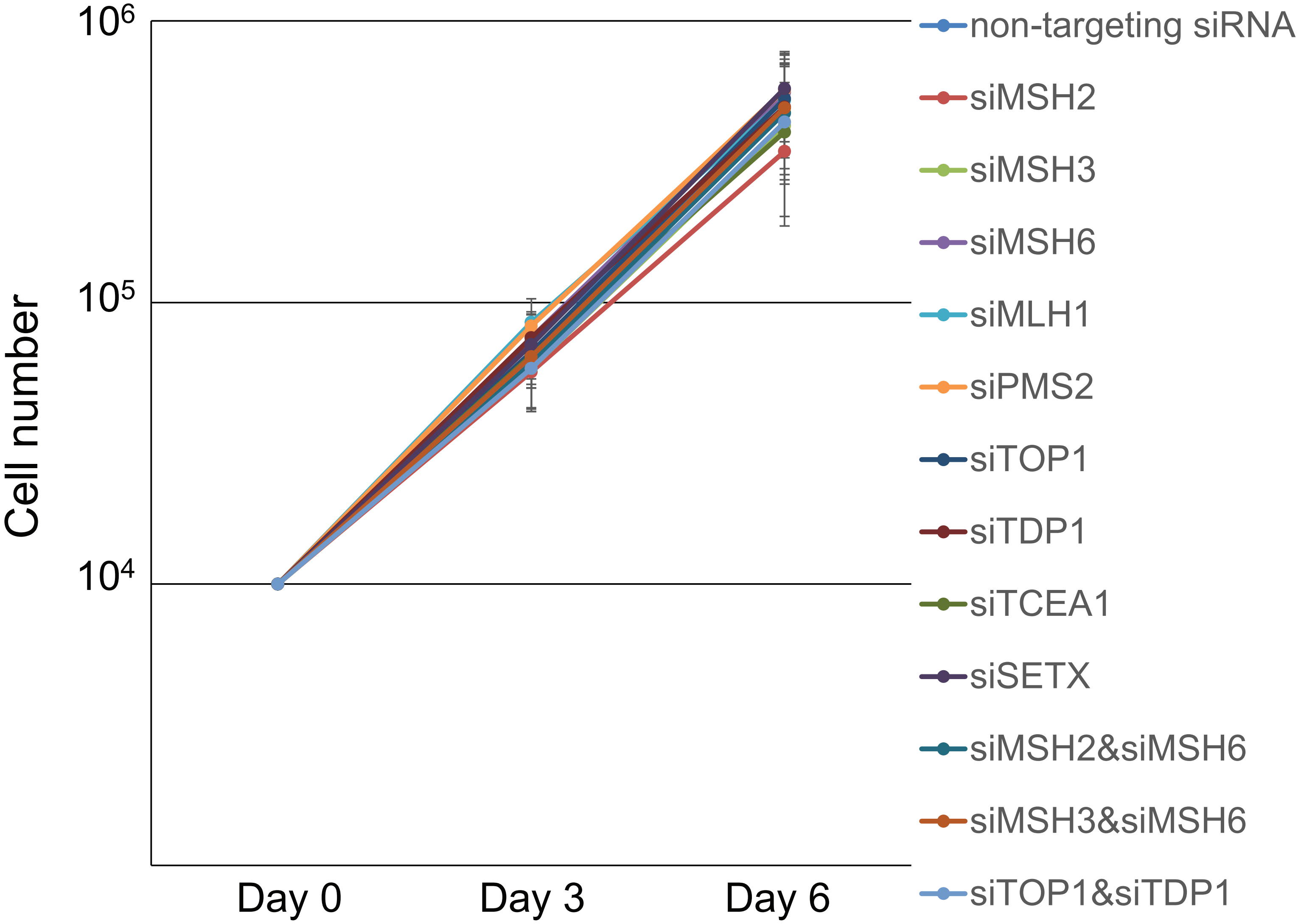


**Supplementary Figure S1.** Growth curves of HT1080-800R cells treated with each siRNA. Error bars indicate the SD of triplicate experiments.

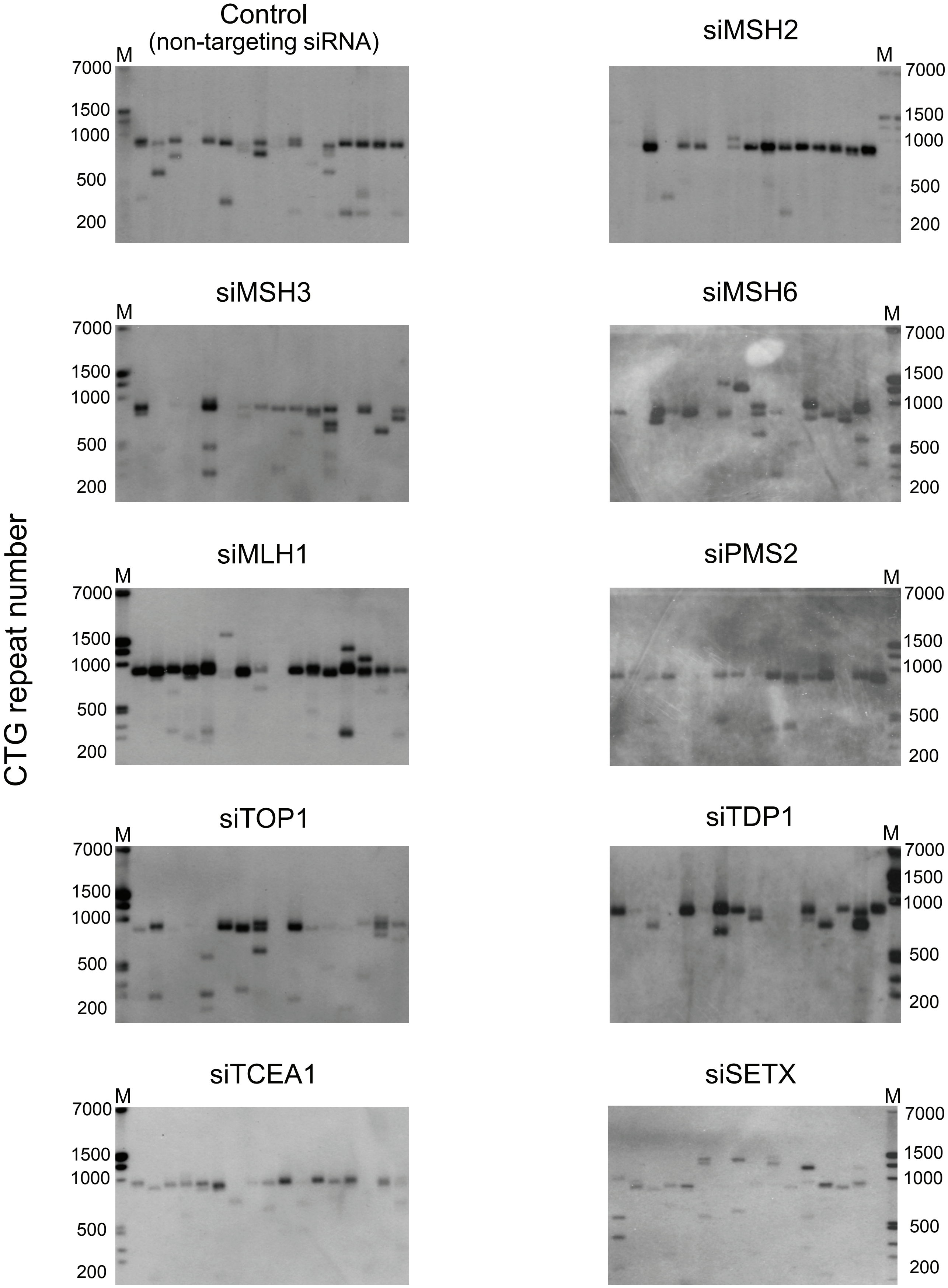


**Supplementary Figure S2.** Small-pool PCR analysis of repeat length in HT1080-800R cells. Representative lanes of small-pool PCR are shown. The scale shows the molecular weight markers (M) converted into numbers of CTG repeats.

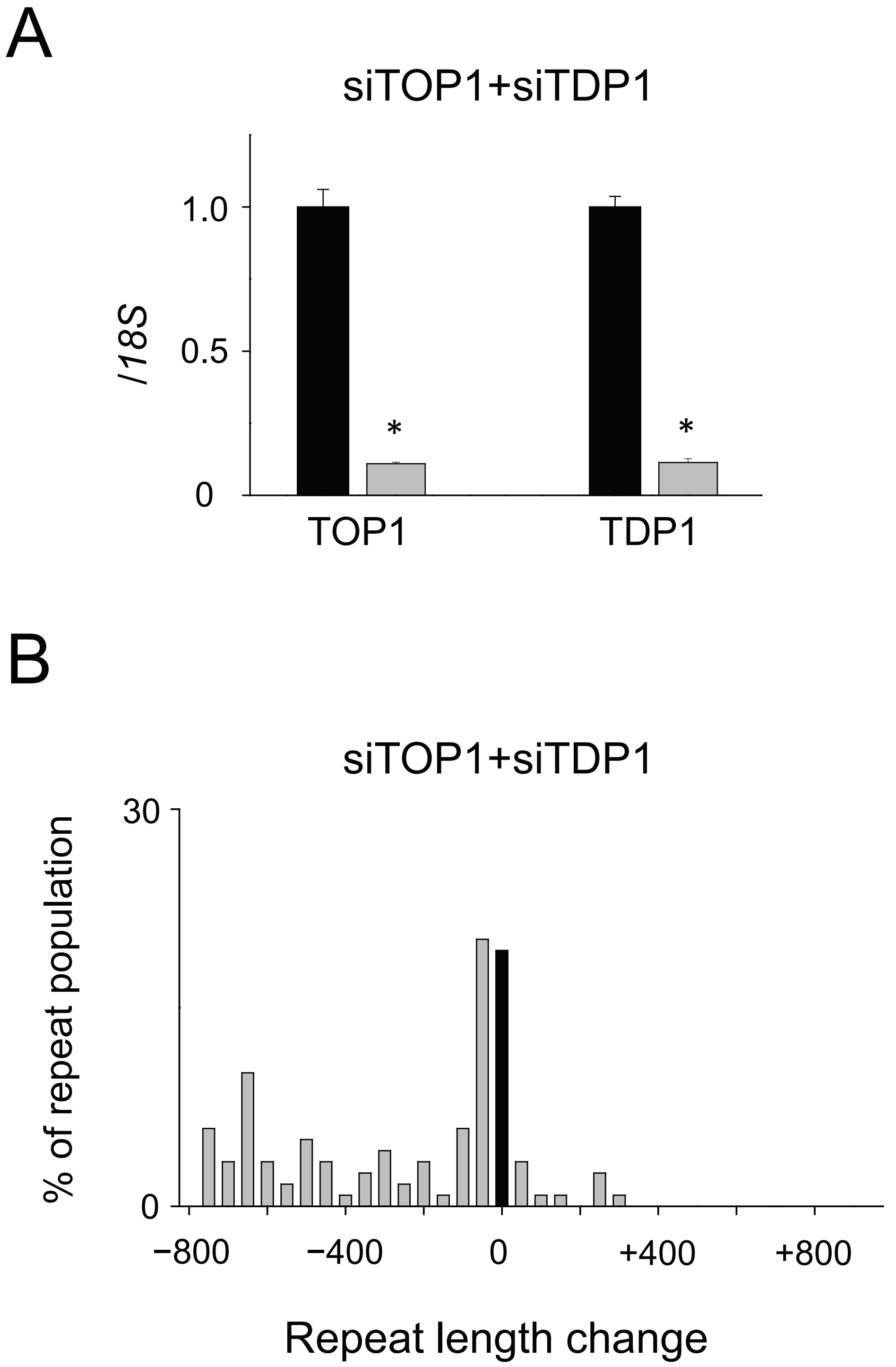


**Supplementary Figure S3.** (A) Expression levels of *TOP1* and *TDP1* following double siRNA knockdown as determined by *18S* rRNA-normalized quantitative reverse transcription PCR. The expression of each target was reduced by sustained specific siRNA knockdown (gray bars) when compared with the expression in cells treated with the non-targeting control siRNA (black bars). Data are presented as means ± standard deviations (SD) of triplicate experiments. *$P < 0.001$.
(B) Effects of double TOP1 and TDP1 knockdown on CTG•CAG repeat instability in HT1080-800R cells. Histograms show the repeat-length distributions in HT1080-800R cells. The frequency distribution of unstable alleles is indicated as gray bars. The frequency of stable alleles is indicated as black bars. Allele lengths are grouped in bins spanning 50 repeats. More than 50 alleles were sized per group.

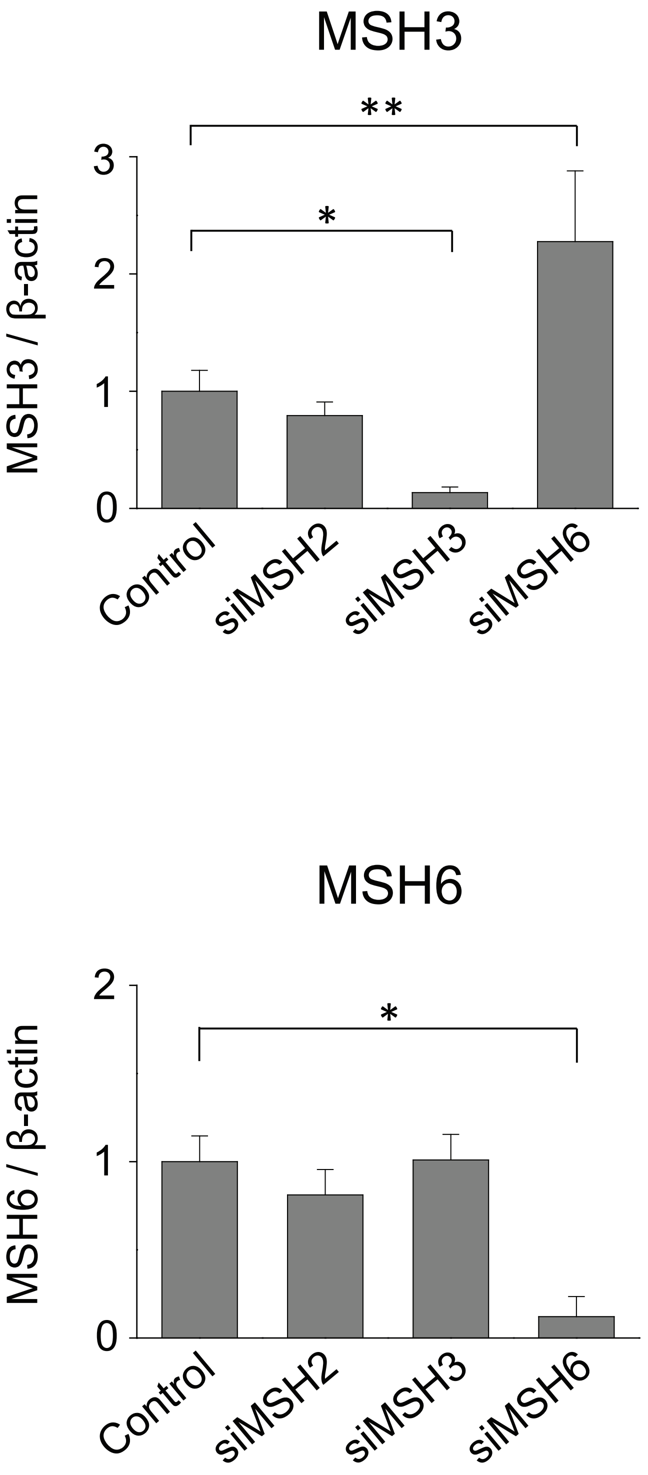


**Supplementary Figure S4.** The relative amounts of MSH3 and MSH6 proteins following each siRNA treatment. Data are presented as means ± SD of triplicate experiments. *$P < 0.05$.

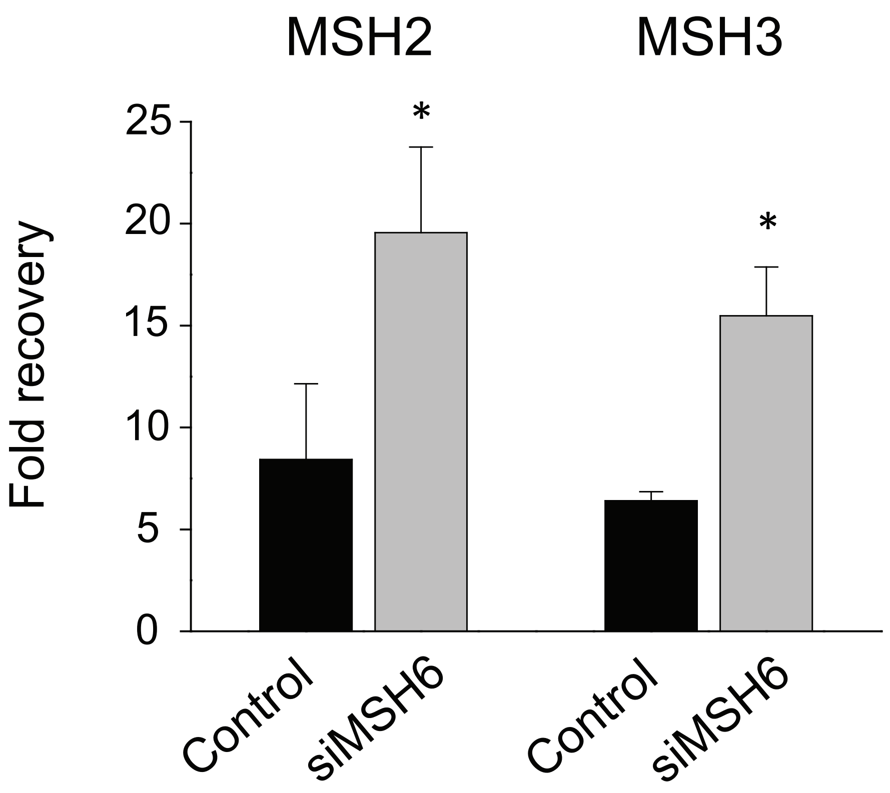


**Supplementary Figure S5**. MSH2 and MSH3 enrichment at the CTG•CAG repeat tract following MSH6 knockdown in human HT1080-800R cells. Chromatin immunoprecipitation assays were performed using MSH2 or MSH3-specific antibodies, and background signals were assessed using normal mouse IgG. Qualitative PCR data indicating MSH2 and MSH3 occupancy are expressed as the fold enrichment over the background signal. Data are presented as means $\pm$ standard deviations of triplicate experiments. *$P$ < 0.05.

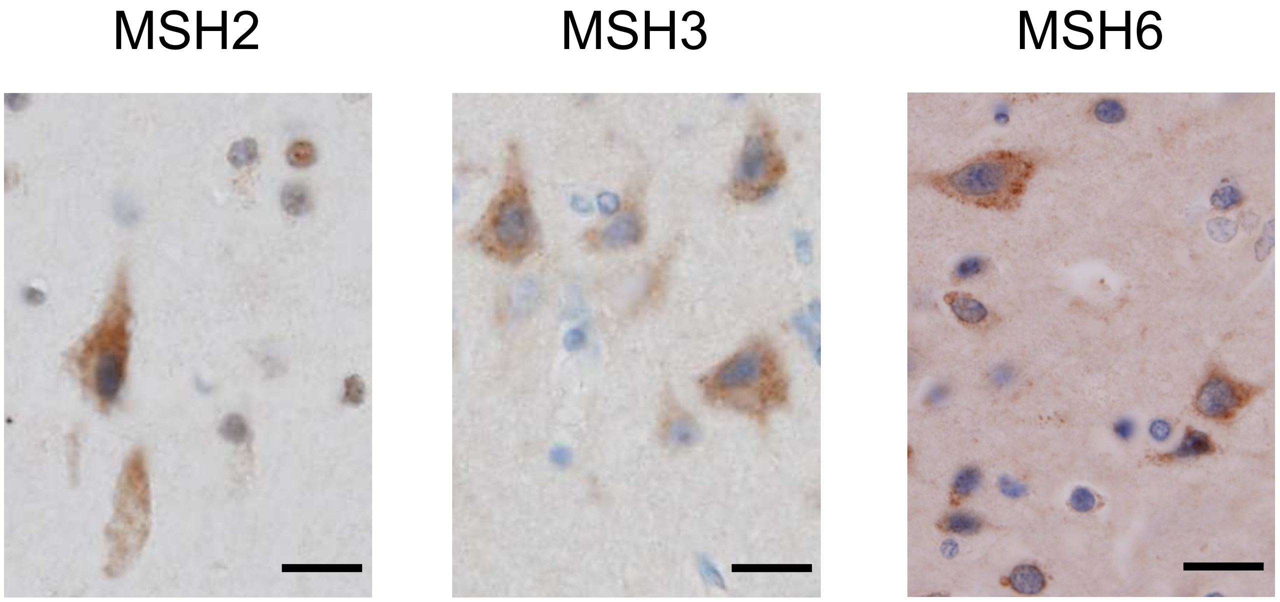


**Supplementary Figure S6**. Immunohistochemistry for MSH2, MSH3, and MSH6 proteins in human brain (temporal lobe). Sections were stained with antibodies to MSH2 (1:200, FE11; Life Technologies), MSH3 (1:100, 611390; BD Biosciences), and MSH6 (1:400, 610919; BD Biosciences). Nuclei were counterstained with haematoxylin. Scale bar: 20 µm.
